# Effects of resveratrol on growth performance, immune function, and intestinal microbiota in Cherry Valley ducks

**DOI:** 10.3389/fmicb.2025.1689994

**Published:** 2025-11-27

**Authors:** Tiantian Gu, Yu Wang, Yan Chen, Jian Li, Shiyu Wang, Yonghai Wang, Xintong Li, Chunyang Wang, Fayi Xiao

**Affiliations:** 1 Department of Veterinary Medicine, Shandong Vocational Animal Science and Veterinary College, Weifang, China; 2 Animal Science and Veterinary Technical Center of Linqu County, Weifang, China; 3 Animal Science and Veterinary Technical Center of Gaomi City, Weifang, China; 4 College of Animal Science and Technology, Shandong Agricultural University, Taian, China

**Keywords:** Cherry Valley duck, resveratrol, growth performance, immunefunction, intestinal microbiota

## Abstract

**Introduction:**

This study investigated the effects of stage-specific supplementation with 0.1% resveratrol on growth performance, immune function, and intestinal microbiota in Cherry Valley ducks from 1 to 42 days of age.

**Methods:**

A total of 300 1-day-old Cherry Valley ducks were randomly allocated into a control group (C) and four treatment groups (R1~R4). Resveratrol was administered via diet at 14, 21, 28, or 35 days of age until 42 days. Growth performance, slaughter traits, serum immune indices, and intestinal microbiota were analyzed.

**Results:**

In growth performance, the R1~R4 groups exhibited significantly improved slaughtering rates compared to the control (*P* < 0.01). The R1&R2 groups showed marked elevations in semi-evisceration rates (*P* < 0.001), while the R1 group displayed a significant increase in full-evisceration rate (*P* < 0.01). In terms of immune function, the R4 group demonstrated elevated serum IgA levels by 46.37%. All treatment groups exhibited upward trends in IgG. Complement C3 in the R2 group was markedly higher than the control (*P* < 0.0001). Interleukins IL-2 and IL4 were significantly upregulated in R1&R2 groups (*P* < 0.01). Thymus and spleen indices showed increasing trends in R3&R4 and R4 groups, respectively. With respect to intestinal microbiota, α-Diversity indices were significantly enhanced in R2&R4 groups (*P* < 0.001). β-Diversity analysis revealed distinct clustering between treated groups and controls. LEfSe analysis identified Bacteroides, Alistipes, and Faecalibacterium as biomarkers in R2&R4 groups, with pathogenic genera (Prevotellaceae, Desulfovibrio) reduced.

**Discussion:**

Resveratrol supplementation improved slaughtering performance, immune competence, and intestinal homeostasis in Cherry Valley ducks. Resveratrol supplementation from day 21 (R2 group) showed the most favorable outcomes for growth-immunity-microbiota axis regulation, highlighting its potential as a precision nutrition strategy in waterfowl aquaculture.

## 1 Introduction

Resveratrol (3,5,4′-trihydroxy-trans-stilbene), a plant-derived natural polyphenolic compound, is widely present in peanuts, Polygonum cuspidatum, grapes, and processed byproducts (Kuršvietiene et al., 2016; Sai-Sai, 2023). Its trans-isomer exhibits superior stability and bioactivity, positioning it as a research hotspot in nutrition and medicine (Canto and Sameer, 2015). Studies demonstrate that resveratrol activates signaling pathways such as SIRT1/Nrf2/ARE, exerting pleiotropic biological functions including immunomodulation, anti-inflammation, anticancer, antioxidation, antimicrobial, and antiviral activities (Alarcon et al., 2004; Alov et al., 2015; Hwang and Lim, 2015; Spanier et al., 2009). In human medicine, resveratrol has shown promise in preventing tumor progression, cardiovascular diseases, and neurodegenerative disorders (Smoliga et al., 2011).

In livestock production, resveratrol serves as a novel functional additive to enhance animal health and productivity. For example, dietary supplementation with 400 mg/kg resveratrol significantly improves the villus height/crypt depth ratio (V/C) in heat-stressed Muscovy ducks, alleviating intestinal inflammation, oxidative stress, and dysbiosis while mitigating deoxynivalenol-induced intestinal barrier damage (He et al., 2019a). Similarly, inclusion of 0.2% resveratrol in pig diets enhances feed conversion efficiency, immunoglobulin G levels, and intestinal immune barrier function by modulating cytokines like IL-6 and TNF-α (Ahmed et al., 2013; Yaolian et al., 2019). Notably, resveratrol exhibits robust antagonistic effects against mycotoxins: it activates the Nrf2 signaling pathway to counteract deoxynivalenol-induced cytotoxicity/apoptosis in porcine intestines and improves pork quality via the lncRNA-KEAP1-NRF2 axis (Yang et al., 2019; Zhang et al., 2024). Collectively, these findings suggest resveratrol exerts multi-target regulatory effects on intestinal health, though its mechanisms in poultry remain poorly elucidated.

Despite extensive research in monogastric species (e.g., pigs, chickens), systematic investigations of resveratrol in aquatic poultry—particularly Cherry Valley ducks—are lacking. Existing literature highlights the breed's vulnerability to rapid fat deposition and diet-induced intestinal dysbiosis (Bai et al., 2024; Li et al., 2023). Recent studies indicate that 400 mg/kg resveratrol improves feed conversion efficiency and modulates anti-inflammatory cytokines (e.g., IL-10, TGF-β) in 21-day-old broilers (Wang et al., 2021). Oral supplementation with Lactobacillus casei, Lactobacillus rhamnosus and Lactobacillus rhamnosus can mitigate avian colibacillosis induced intestinal flora dysbiosis (Shi et al., 2020). Adding 6% paper mulberry powder to the daily diet can enhance the cecal microbial diversity of Cherry Valley ducks and increase the abundance of *Bacteroides* (Xiong et al., 2025). Muscovy duck probiotics fermented feedstuff can promote the growth of core probiotics and reduce potential pathogenic bacteria (Li et al., 2024). However, critical gaps persist regarding optimal supplementation timing, dose-response relationships, and synergistic effects on the gut-immune axis for Cherry Valley ducks.

Therefore, this study evaluated the effects of stage-specific resveratrol supplementation (0.1%) on growth performance, immune function, and intestinal microbiota in Cherry Valley ducks. The objectives were to identify the optimal intervention window for improving intestinal health and provide a theoretical foundation for the application of resveratrol in precision nutrition strategies for waterfowl. Furthermore, these findings aim to facilitate the practical implementation of resveratrol as an antibiotic alternative in sustainable aquaculture practices.

## 2 Materials and methods

### 2.1 Experimental reagents

Resveratrol (0.1% premixed feed) was purchased from Shandong Kangpushan Biotechnology Co., Ltd. (Product name: Tefumei Livestock and Poultry Compound Premix Feed). The 0.1% dose was selected based on preliminary dose-response trials in ducks and consistent with effective concentrations reported in previous studies (He et al., 2019a; Wang et al., 2021).

### 2.2 Experimental design

A completely randomized single-factor design was adopted. A total of 300 one-day-old healthy commercial Cherry Valley ducks were randomly assigned to five groups (*n* = 6 replicates/group, 10 ducks/replicate): Control group (C): Basal diet without resveratrol. Treatment groups (R1~R4): Basal diet supplemented with 0.1% resveratrol starting at 14, 21, 28, or 35 days of age, respectively, until 42 days.

Basal Diet Composition (Table 1) was formulated to meet or exceed NRC (2012) nutrient requirements for meat ducks. Daily observations included health status, feed/water intake, fecal abnormalities, and mortality. At 42 days, growth performance, immune indices, serum oxidative status, and intestinal microbiota were analyzed.

**Table 1:** Composition and nutritional levels of the basal diet.

**Components**	**Proportion (%)**	**Nutrient levels**	**Value**
Rough rice and wheat mixture	41.87	Crude protein (%)	18.52
Wheat flour	22.30	Crude fat (%)	8.18
Food residue	8.50	Crude ash (%)	4.17
Secondary lard	3.00	Moisture (%)	12.51
Soybean meal, CP45.0	1.60	Calcium (%)	0.60
Soybean meal, CP46.0	4.20	Total phosphorus (%)	0.61
Peanut cake (flake)	1.00	Metabolizable energy (kcal/kg)	3,289.64
Corn gluten meal	4.00	Lysine (%)	1.19
Distillers dried grains with solubles (DDGS)	2.00	–	–
Corn sugar residue	1.00	–	–
Chicken hydrolyzed feather meal	1.00	–	–
Pork meal	4.00	–	–
Chicken powder	1.50	–	–
Monosodium glutamate residue	1.50	–	–
L-Lysine sulfate, 70%	0.91	–	–
Premix^*^	1.62	–	–

^*^Each kilogram of premix contains: Vitamins: Vitamin A: 60 KIU Vitamin B1 (Thiamine): ≥15 mg, Vitamin B_2_ (Riboflavin): ≥30 mg, Pantothenic acid: ≥150 mg, Vitamin B6 (Pyridoxine): ≥10 mg, Vitamin B1_2_ (Cobalamin): ≥0.2 mg, Vitamin D3: 40 KIU, Vitamin E: ≥150 KIU, Vitamin K3: ≥25 mg, Folic acid: ≥8 mg, Niacin (Nicotinic acid): ≥200 mg, Choline chloride: ≥3000 mg. Minerals: Copper (Cu): ≥500 mg, Iron (Fe): ≥1500 mg, Manganese (Mn): ≥900 mg, Zinc (Zn): ≥1250 mg, Iodine (I): ≥3 mg, Selenium (Se): ≥20 mg.

### 2.3 Animal management

Cherry Valley ducks were raised under a raised wire flooring system with each pen serving as a replicate unit. Environmental conditions (temperature, humidity, and ventilation) were standardized across pens. A continuous lighting regime with an intensity of 5 lux was implemented for 24 h to maintain photoperiod consistency. Mechanical ventilation was employed to ensure air quality. Immunization protocols followed the National Technical Code for Commercial Meat Duck Production (GB/T 22346–2008). Daily records included ambient temperature/humidity, weather conditions, and observations of feeding behavior, water intake, and flock health status.

### 2.4 Measurement indices and methods

#### 2.4.1 Growth performance

Body weight was recorded at day 42 (fasted overnight starting at 08:00). Growth parameters were calculated as follows: Average Daily Gain (ADG, g/bird/day) = Total weight gain/Experimental days. Average Daily Feed Intake (ADFI, g/bird/day) = Total feed consumption/Experimental days. Feed-to-Gain Ratio (F/G) = ADFI/ADG.

#### 2.4.2 Slaughter traits

In the experiment, Cherry Valley ducks received an intravenous injection of 50 mg/kg pentobarbital sodium. Following anesthesia induction, the carotid artery was transected to induce exsanguination, resulting in cardiac arrest. Five birds per group were randomly selected for slaughter according to NY/T 823–2004 (Terminology and Metrics for Poultry Production Performance). Traits measured included: Slaughter Rate (%) = (Carcass weight/Live weight) × 100. Evisceration Rates: Semi-evisceration Rate (%) = (Semi-eviscerated weight/Carcass weight) × 100, Full-evisceration Rate (%) = (Full-eviscerated weight/Carcass weight) × 100. Muscle Yields: Breast muscle yield (%) = (Breast meat weight/Full-eviscerated weight) × 100, Leg muscle yield (%) = (Leg meat weight/Full-eviscerated weight) × 100.

#### 2.4.3 Immune organ indices

Five birds per group were euthanized for immune organ collection. Organ indices were calculated as: Thymus Index (mg/g) = Thymus weight/Live body weight, Spleen Index (mg/g) = Spleen weight/Live body weight, Bursa of Fabricius Index (mg/g) = Bursa weight/Live body weight.

#### 2.4.4 Serum immune indices assay

Blood samples were collected via wing vein puncture into heparinized tubes. Plasma was separated by centrifugation at 3,000 × g for 15 min at 4 °C. Concentrations of IgA, IgG, IgM, complement components C3/C4, and cytokines IL-2, IL-4, IL-6, and IFN-γ were quantified using enzyme-linked immunosorbent assays (ELISA) following the manufacturer's instructions. Kits for all analytes were purchased from Beijing Huaying Biotechnology Co., Ltd. (Beijing, China). Absorbance was read at 450 nm using a microplate reader (BioTek Instruments, Winooski, VT, USA).

#### 2.4.5 Intestinal microbiota high-throughput sequencing

Select 5 ducks per group, aseptically collect 1.0 g cecal contents into 2 mL sterilized cryovials, rapidly preserve in liquid nitrogen, then transfer to −80 °C freezer for sequencing. Extract genomic DNA using the CTAB method. Amplify the V3-V4 region of the 16S rRNA gene via PCR. Perform sequencing on the Illumina NovaSeq platform. Cluster PCR products and purify. Complete library construction and sequencing by NovoMagic Co., Ltd. Use QIIME2 software for ASV clustering (97% similarity). Alpha diversity (Shannon index, Chao1 index) was calculated using QIIME2. Beta diversity was assessed via principal coordinate analysis (PCoA) based on Bray-Curtis dissimilarity matrices. Differential abundance analysis was performed using the linear discriminant analysis (LDA) effect size (LEfSe) algorithm (LDA score > 4.0).

### 2.5 Data statistics

Experimental data were organized using Excel software. Statistical analysis was performed with SPSS 20.0 software via analysis of variance (ANOVA). Normality and homoscedasticity were verified using Shapiro-Wilk and Levene's tests, respectively, prior to ANOVA. Intergroup significance was determined by Duncan's multiple range test (DMRT) with a significance threshold of *P* < 0.05. Results are presented as mean ± standard deviation.

## 3 Result

### 3.1 Effects of resveratrol on different ages on growth performance

As shown in Table 2, no statistically significant differences were observed in average daily gain (ADG) or average daily feed intake (ADFI) across all groups during the entire 1~6 weeks growth period (*P* > 0.05). Notably, a specific trend emerged in feed-to-gain ratio (F/G) at week 6, where the R1 group exhibited a significantly lower F/G value (2.69 ± 0.05) compared to control groups (2.99 ± 0.01) (*P* < 0.05). As a critical indicator of feed conversion efficiency, this result suggests that resveratrol supplementation may optimize nutrient metabolism in late-stage growth stages, thereby enhancing dietary utilization efficiency in Cherry Valley ducks.

**Table 2:** Effect of resveratrol on the growth performance.

**Weeks**	**Groups**	**ADG (g/d)**	**ADFI (g/d)**	**F/G**
1	C	30.14 ± 2.60	32.65 ± 2.50	1.08 ± 0.05^ab^
	R1	30.00 ± 2.80	31.98 ± 2.72	1.08 ± 0.03^ab^
	R2	28.57 ± 3.05^a^	30.64 ± 2.65	1.07 ± 0.04^b^
	R3	29.29 ± 2.65^b^	30.97 ± 2.81	1.06 ± 0.07^a^
	R4	30.71 ± 2.74^a^	33.01 ± 2.66	1.07 ± 0.05^ab^
2	C	66.35 ± 2.45	86.47 ± 4.52	1.30 ± 0.05^ab^
	R1	67.86 ± 2.90	88.49 ± 4.04	1.30 ± 0.06^ab^
	R2	67.14 ± 2.84	86.03 ± 3.36	1.28 ± 0.05^b^
	R3	65.71 ± 2.78	85.21 ± 4.40	1.29 ± 0.04^b^
	R4	65.00 ± 2.69	85.03 ± 5.92	1.31 ± 0.04^a^
3	C	90.31 ± 3.03^a^	162.85 ± 4.85^b^	1.80 ± 0.04^b^
	R1	89.29 ± 3.02^a^	159.84 ± 6.23	1.79 ± 0.05^b^
	R2	89.29 ± 2.91^b^	160.87 ± 5.55^b^	1.80 ± 0.03^a^
	R3	102.14 ± 3.11^b^	182.30 ± 5.62	1.78 ± 0.03^ab^
	R4	94.29 ± 2.89^a^	172.72 ± 6.03	1.83 ± 0.04^b^
4	C	127.86 ± 2.45	202.54 ± 18.25	1.58 ± 0.03^a^
	R1	157.14 ± 3.07	216.51 ± 17.77	1.38 ± 0.06^ab^
	R2	157.86 ± 2.94	212.38 ± 16.43	1.35 ± 0.07^b^
	R3	142.85 ± 2.88	209.52 ± 18.92	1.47 ± 0.06^ab^
	R4	140.00 ± 2.92	211.90 ± 18.34	1.51 ± 0.07^a^
5	C	126.43 ± 2.53	241.90 ± 22.65	1.91 ± 0.03^a^
	R1	127.86 ± 2.42	245.40 ± 22.45	1.92 ± 0.04^a^
	R2	130.00 ± 2.88	244.76 ± 23.05	1.88 ± 0.03^a^
	R3	121.43 ± 2.23	247.62 ± 22.77	2.04 ± 0.05^a^
	R4	136.43 ± 2.43	244.44 ± 22.69	1.79 ± 0.06^a^
6	C	69.64 ± 2.45^ab^	208.32 ± 18.27	2.99 ± 0.01^a^
	R1	76.43 ± 2.17^ab^	206.51 ± 17.47	2.69 ± 0.05^b^
	R2	68.57 ± 2.36^a^	202.38 ± 16.42	2.95 ± 0.01^a^
	R3	67.14 ± 2.45^a^	199.52 ± 18.62	2.97 ± 0.02^a^
	R4	71.43 ± 2.11^ab^	211.90 ± 18.64	2.96 ± 0.05^a^

Different letters of the shoulder label in the same column indicate significant differences (*P* < 0.05), and the same letters or no letter labels of the shoulder labels indicate no significant differences (*P* < 0.05). The table below is the same.

### 3.2 Effects of resveratrol on slaughter performance

**Figure 1:**
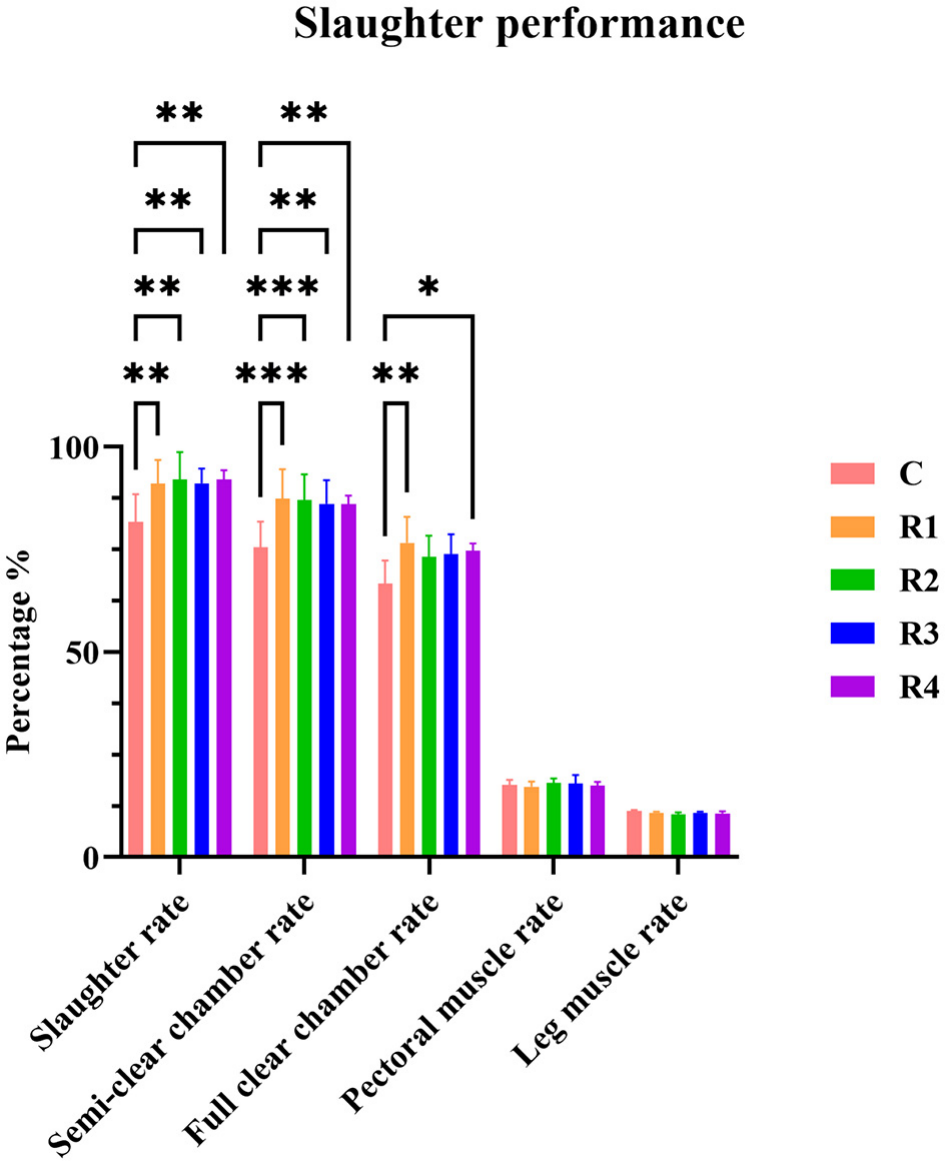
Effects of resveratrol on slaughter performance in cherry valley ducks. *P*-values are typically indicated by asterisks (*) to denote significance levels. Different numbers of asterisks represent different *P*-value ranges and significance levels: *P* > 0.05: Not significant (ns), *P* ≤ 0.05: Significant, denoted by one asterisk (*), *P* ≤ 0.01: Highly significant, denoted by two asterisks (**), *P* ≤ 0.001: Extremely significant, denoted by three asterisks (***).

Comparison between the control group and resveratrol-treated groups (R1~R4) revealed that resveratrol significantly improved slaughter performance (Figure 1). Slaughter rate was markedly increased in R1~R4 groups (*P* < 0.01 vs. C group). Semi-evisceration rate showed extreme significance in R1&R2 groups (*P* < 0.001) and significance in R3&R4 groups (*P* < 0.01). Full-evisceration rate was significantly higher in R1 (*P* < 0.01) and R4 (*P* < 0.05) groups. No significant differences were observed in breast muscle or leg muscle rates (*P* > 0.05), indicating resveratrol effectively enhances slaughter performance without affecting muscle mass distribution.

### 3.3 Effects of resveratrol on immune organ indices

**Figure 2:**
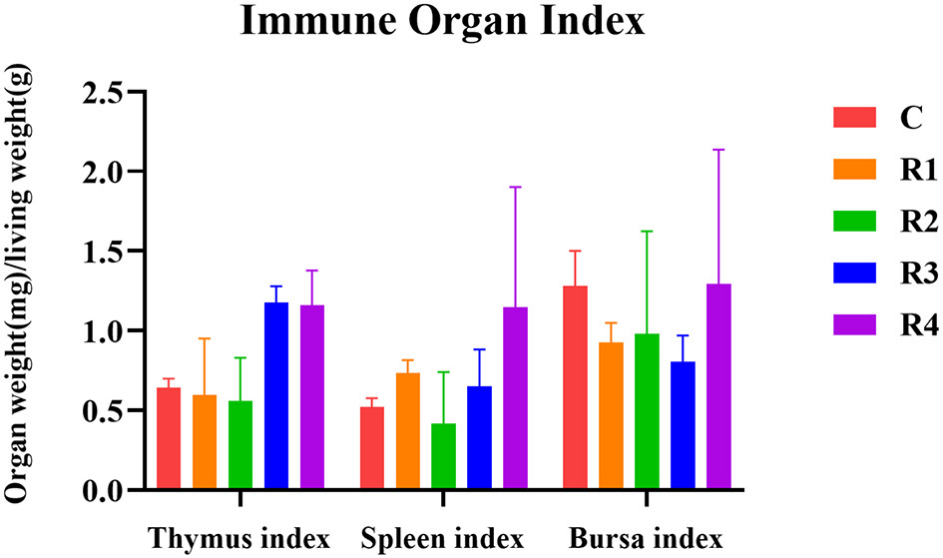
Effect of resveratrol on immune organ index.

The regulatory effects of resveratrol on immune organ development were demonstrated that R3 and R4 groups (1.18 ± 0.10 mg/g, 1.16 ± 0.22 mg/g) showed slight increases compared to C group (0.64 ± 0.06 mg/g, *P* >0.05) in thymus index (Figure 2). About spleen index, R4 group reached 1.15 ± 0.75 mg/g (vs. 0.52 ± 0.05mg/g in C group, *P* > 0.05), suggesting positive regulation. No significant differences among groups (*P* > 0.05) of bursa fabricius index, indicating limited impact.

### 3.4 Effects of resveratrol on serum immune indicators

**Figure 3:**
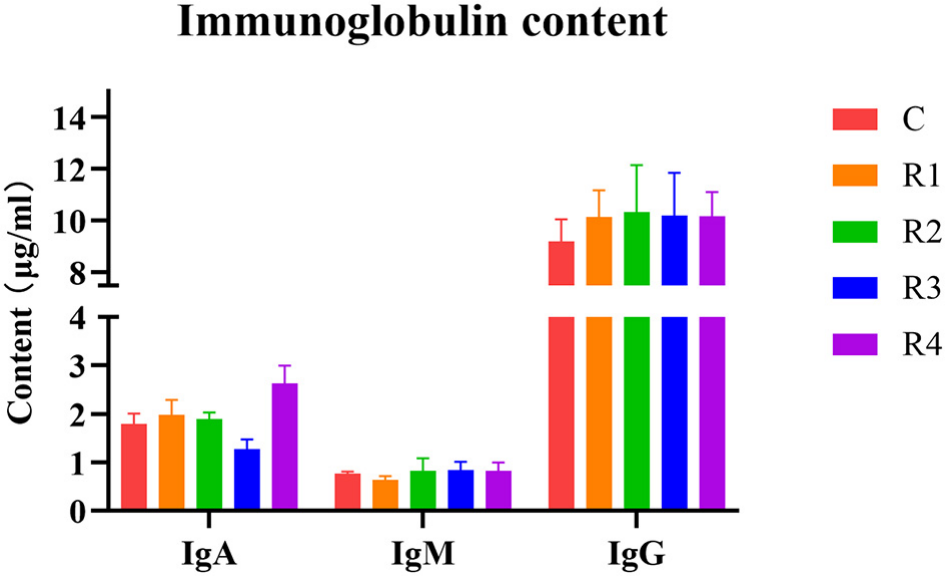
Effect of resveratrol on immunoglobulin.

Analysis of serum immunoglobulins (Figure 3) showed that IgA content significantly increased 46.37% in R4 group (vs. C group), IgM showed no significant differences, and IgG exhibited an increasing trend in R1-R4 groups (*P* > 0.05). These results demonstrate resveratrol's ability to upregulate IgA and IgG levels, enhancing humoral immunity.

**Figure 4:**
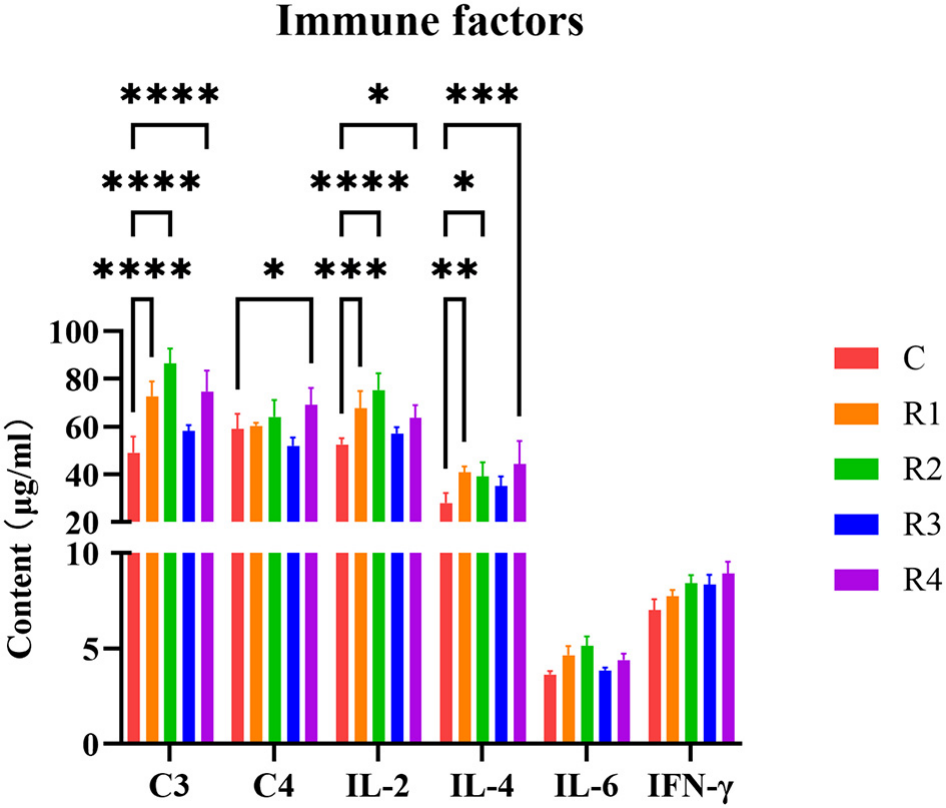
Effect of resveratrol on immune factors. In this context, the asterisks (*) are used to indicate the probability value (*p*-value) of a statistical test, *p < 0.05, This is the most common threshold; **p < 0.01, Stronger significance; ***p < 0.001, Very strong significance. ****p < 0.0001, Extremely strong significance.

For immune factors, complement C3 was extremely significantly higher in R1, R2 and R4 groups (*P* < 0.0001), Compared with the control group, the increases were 48.35%, 76.10%, and 52.39%, respectively (Figure 4). A significant increase in C4 levels was observed in group R4 compared to the control group (17.13%; *P* < 0.05). For Cytokines, IL-2 was extremely significantly elevated in R1 and R2 groups (28.86% and 43.19%, *P* < 0.001); IL-4 significantly higher in R1, R2 and R4 groups (46.21%, 39.96% and 58.52%, *P* < 0.05); and IL-6 and IFN-γ showed increasing trends (*P* >0.05). These findings indicate resveratrol effectively regulates immune factors, particularly enhancing complement and cytokine responses in R1 and R2 groups.

### 3.5 Effects of resveratrol on intestinal microbiota

#### 3.5.1 ASV analysis of intestinal microbiota

**Figure 5:**
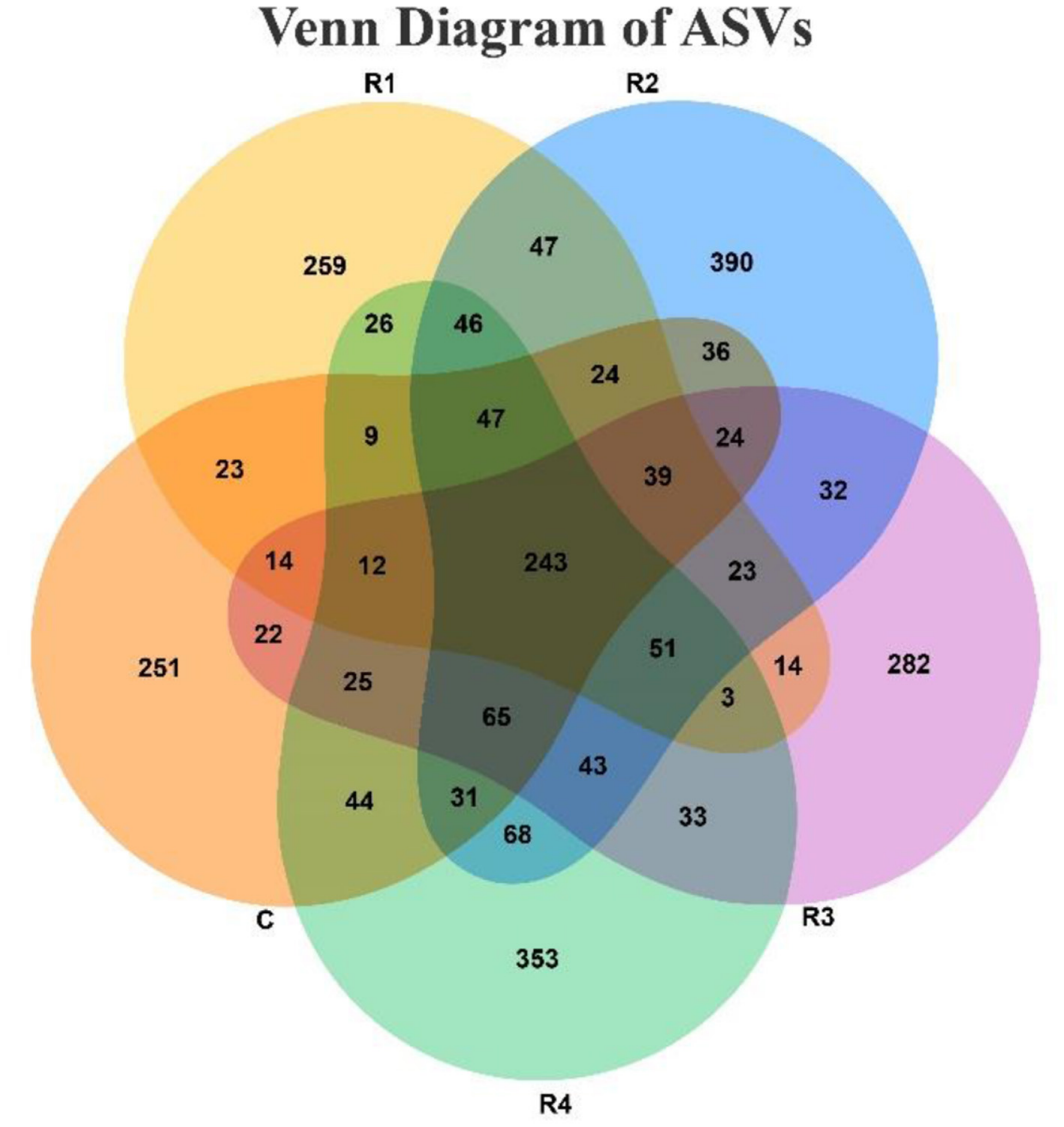
Characteristics of ASV distribution between groups in Venn diagram. Each circle represents a group sample. Numbers in overlapping regions between circles indicate the number of shared ASVs between samples (groups), while numbers in non-overlapping regions indicate group-specific ASVs.

The Venn diagram (Figure 5) clearly illustrates that resveratrol treatment significantly altered the microbial community structure. A total of 243 shared ASVs were detected across all five groups, indicating the presence of a core microbiota prevalent in the gut. However, each treatment group (R1~R4) developed distinct microbial compositions, as reflected by the number of group-specific ASVs (C: 251; R1: 259; R2: 390; R3: 282; R4: 353). Moreover, the number of shared ASVs between groups varied: only 411 ASVs were shared between the C and R1 groups, whereas 594 ASVs were shared between the R2 and R4 groups. The R2, R3 and R4 cluster exhibited strong community associations, sharing 402 ASVs, whereas the C, R1 and R4 cluster showed weaker associations with only 311 shared ASVs. These differences in sharing patterns, combined with the presence of group-specific ASVs, collectively indicate that resveratrol and varying treatment durations exerted selective pressures on the microbiota, shaping group-specific microbial communities.

#### 3.5.2 Effects of resveratrol on intestinal microbial abundance

**Figure 6:**
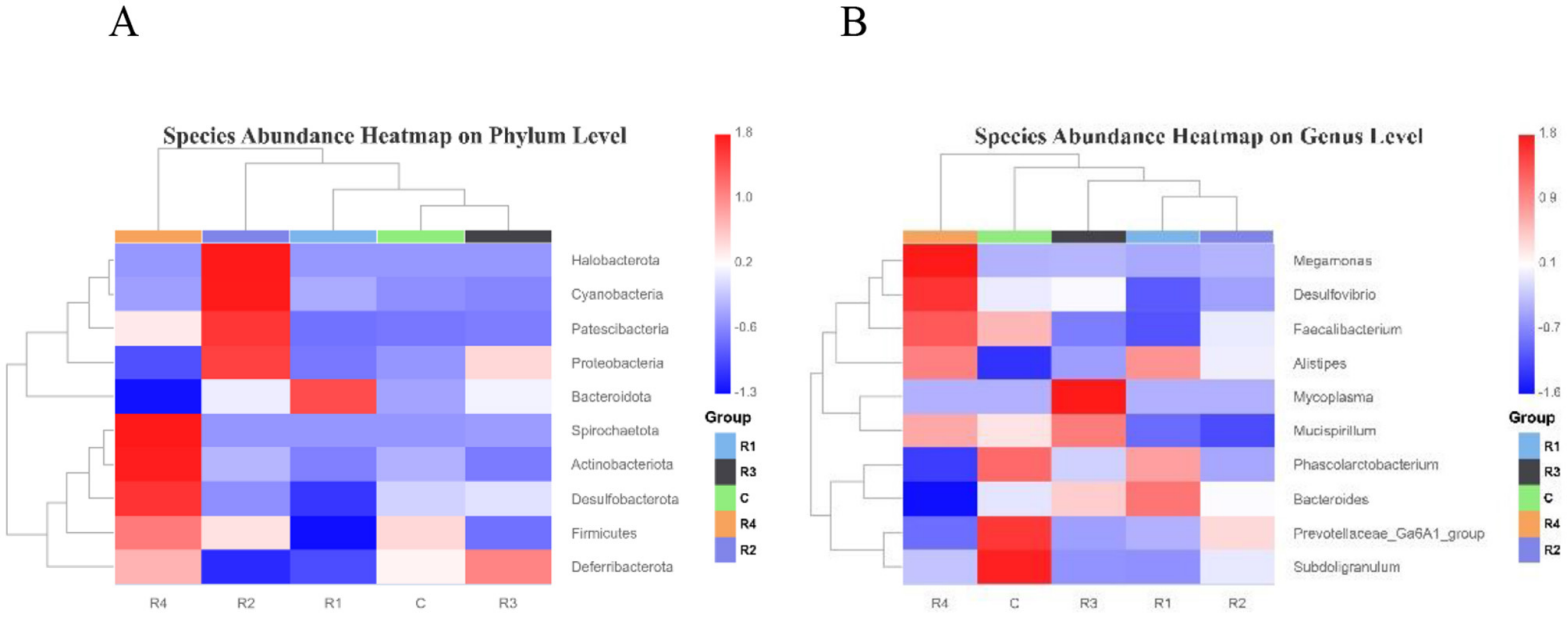
Heatmap of intestinal microbial abundance on Phylum level **(A)** and Genus level **(B)**. Top 10 species (phylum/genus) selected by abundance; heatmap from species/group clustering, color intensity (red = high, blue = low) reflects relative abundance.

The heatmap of species abundance clustering (Figure 6) visually illustrates the differential relative abundances of intestinal microbiota at the phylum and genus levels across resveratrol-treated groups.

At the genus level, the control group showed significant enrichment of *Prevotellaceae, Subdoligranulum*, and *Phascolarctobacterium*, while *Alisipes* abundance was relatively low. R1 group elevated *Bacteroides* abundance, with significant reductions in *Desulfovibrio* and *Faecalibacterium*, R3 group increased *Mycoplasma* and *Mucispirillum* abundances, and R4 group with higher levels of *Megamonas, Desulfovibrio*, and *Alisipes*. Notably, *Prevotellaceae* and *Subdoligranulum* abundances were significantly reduced in R1, R3, and R4 groups compared to the control.

At the phylum level, R1 group enriched *Bacteroidota*, but depleted *Desulfobacterota, Firmicutes*, and *Deferribacterota*. R2 group *elevated Halobacterota, Cyanobacteria, Patescibacteria*, and *Proteobacteria*, and R3 group Increased *Deferribacterota* abundance, however R4 group has higher *Spirochaetota, Actinobacteriota*, and *Desulfobacterota*, but reduced *Bacteroidota*. Resveratrol administration significantly reshaped the gut microbial community, with distinct temporal effects: R1 group characterized by *Bacteroidota* expansion and Firmicutes reduction. R4 group showed a unique restructuring pattern with elevated *Spirochaetota* and *Actinobacteriota*, alongside suppressed *Bacteroidota*.

#### 3.5.3 Effects of resveratrol on alpha diversity of intestinal microbiota

**Figure 7:**
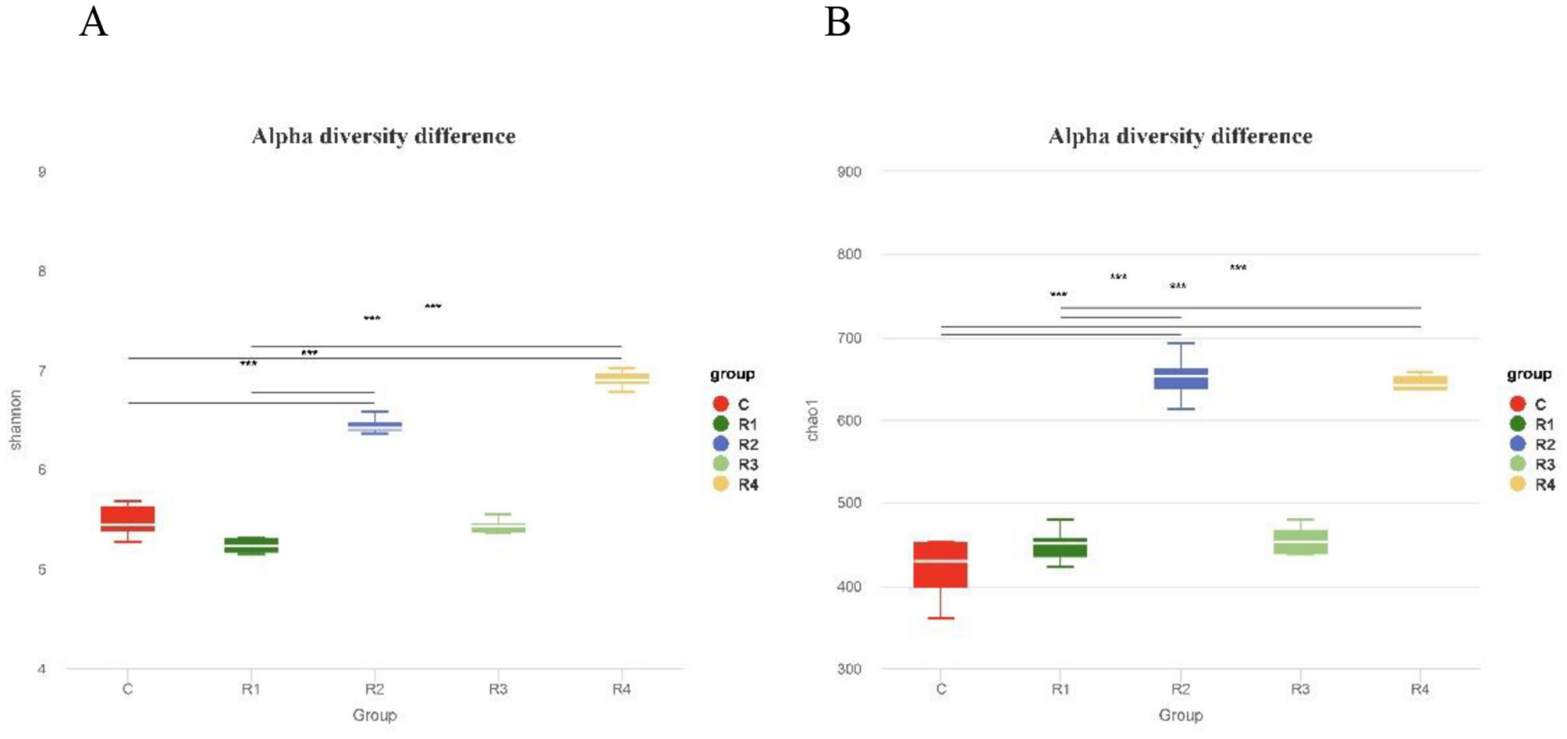
Shannon index **(A)** and Chao1 index **(B)** of intestinal microbiota α diversity. Boxplots show intra-group diversity metrics (median, dispersion, max/min, outliers). Chao1: ASV count (higher = richer). Shannon: Richness/evenness (higher = more diverse/even). Tukey test: Compares diversity differences. Significance: *P* < 0.01 (extremely significant), *P* < 0.05 (significant), *P* > 0.05 (no).

Alpha diversity analysis (Figure 7) revealed that both Chao1 index (reflecting community richness) and Shannon index (reflecting community diversity) were significantly higher in R2 and R4 groups compared to the control group (*P* < 0.001). These findings indicate that resveratrol effectively enhanced the richness and diversity of intestinal microbiota, with the R2 and R4 groups exhibiting the most pronounced effects.

#### 3.5.4 Effects of resveratrol on beta diversity of intestinal microbiota

**Figure 8:**
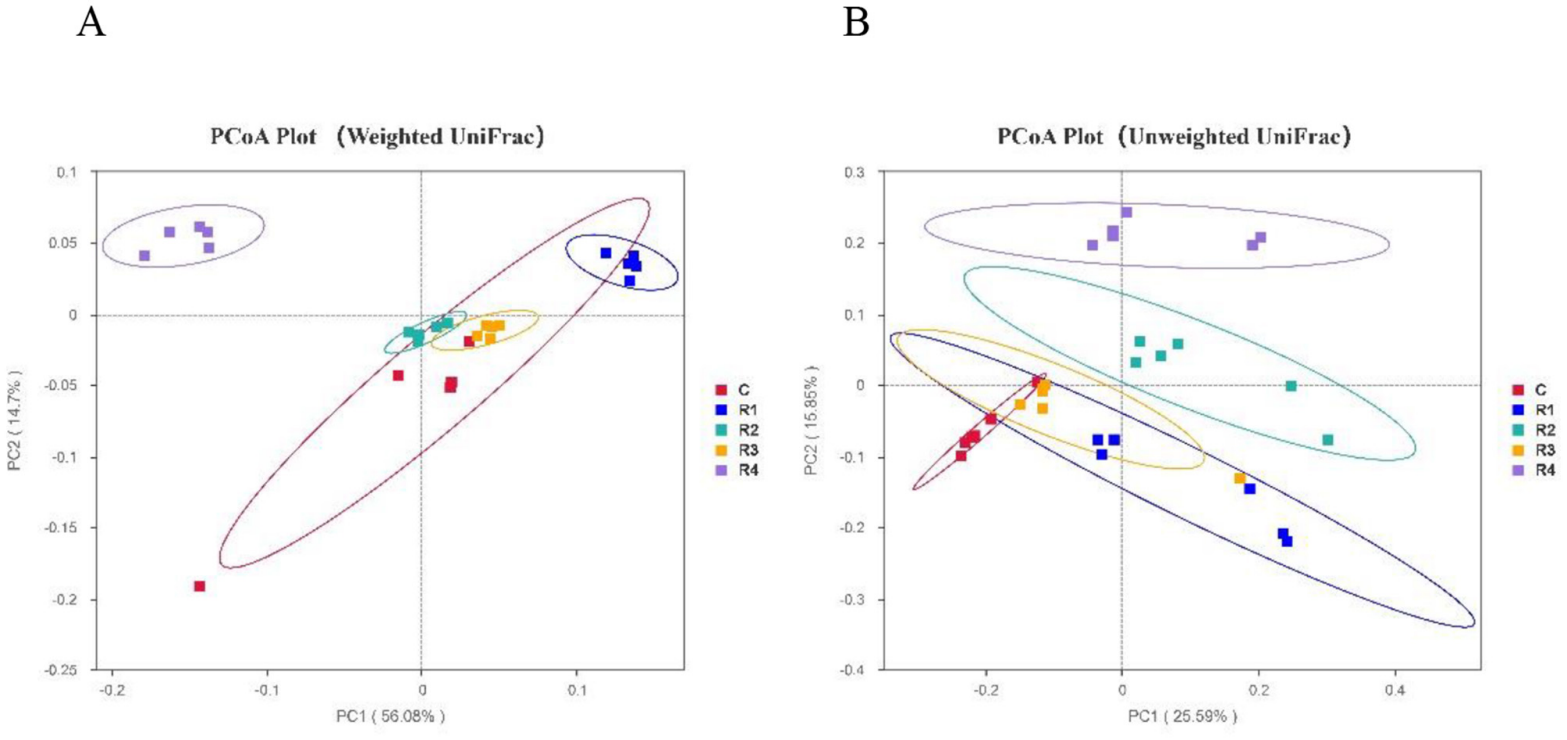
PCoA of intestinal microbiota β diversity. Beta diversity reflects microbiota composition/distribution differences among samples. Dots (samples, color-coded by group): closer = similar structure; farther = larger differences. **(A)** Weighted UniFrac-PCoA; **(B)** Unweighted UniFrac-PCoA.

Beta diversity analysis based on Weighted UniFrac distances (Figure 8) demonstrated significant differentiation in microbial community composition between resveratrol-treated groups (R1, R2 and R4) and the control group. Notably, the R4 group showed the most distinct divergence from all other groups (*P* < 0.01). Further analysis using Unweighted UniFrac distances revealed clear clustering distinctions between R4, R2, R1 groups and the control (*P* < 0.001). These results suggest that resveratrol not only reshaped the overall distribution of gut microbial species (Weighted UniFrac) but also modulated the relative abundance of key taxa (Unweighted UniFrac), achieving targeted interventions in the gut microecological structure of Cherry Valley ducks.

#### 3.5.5 Analysis of significantly differential intestinal microbiota

**Figure 9:**
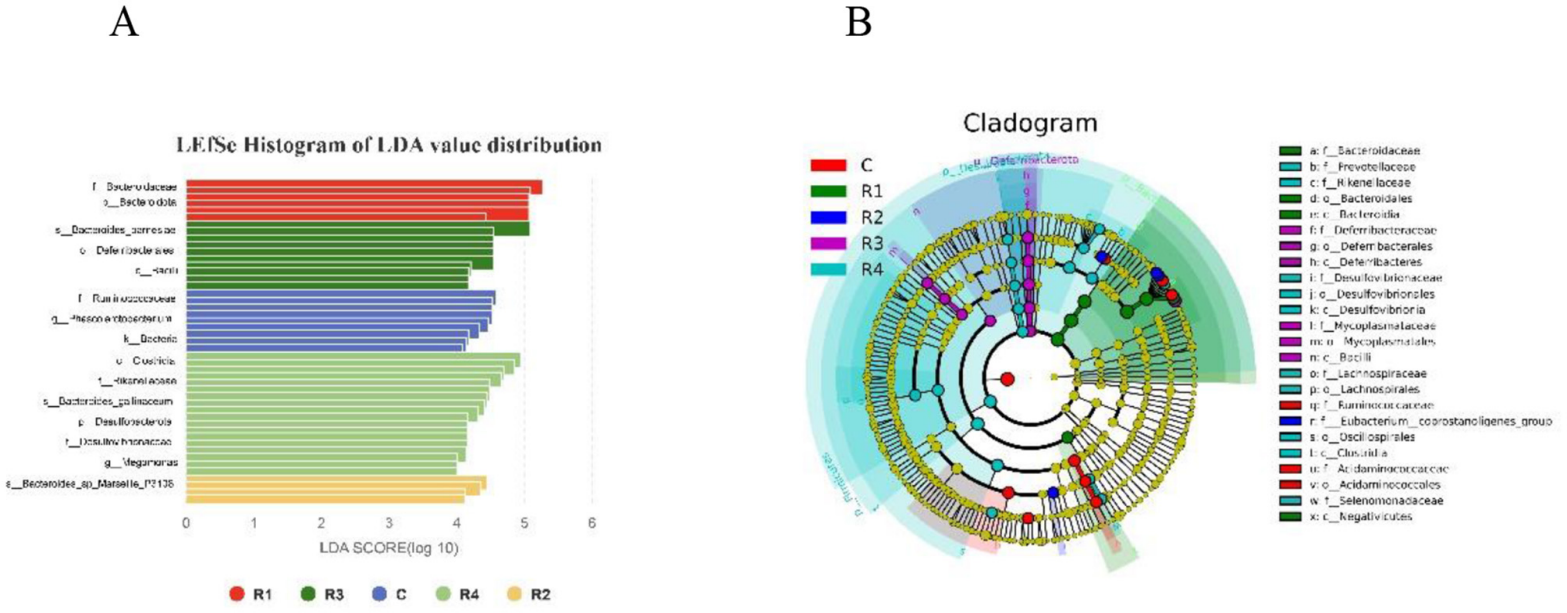
Significant differential microflora in the intestinal microbiota with LEfSe Histogram **(A)** and Cladogram **(B)**. LEfSe results include: LDA histogram (shows significant species; x = LDA, threshold 4.0; bar color = group, length = LDA magnitude) and phylogenetic tree (concentric circles for 7 ranks; node size = abundance, color = significance: yellow = no difference, red = significant difference with higher abundance).

Using LEfSe analysis (LDA >4.0, *P* < 0.05), we identified multiple groups of statistically significant biomarkers across treatments (Figure 9). Specific findings include: R1 group dominated by *Bacteroidota* (LDA = 5.90), with significant enrichment of the genus *Bacteroides* (e.g., B. *caecigallinarum*, LDA = 5.42). R2 group featured Prevotellaceae_UCG_001 (LDA = 4.12) as a core species, alongside significant enrichment of *Bacteroides_*sp_*Marseille* (LDA = 4.85) and *Eubacterium_coprostanoligenes*_group (LDA = 4.41). R3 group specifically enriched in *Deferribacterota* (LDA= 4.56), with mucosal inflammation-associated Mucispirillum (LDA = 4.92) and Firmicutes Mycoplasma (LDA = 4.50) as key biomarkers. R4 group highlighted by Firmicutes taxa, including butyrate-producing *Faecalibacterium* (LDA = 4.15) and *Selenomonadaceae_Megamo* (LDA = 4.34). Additionally, *Alistipes* spp. (e.g., A. inops, LDA = 4.32, with gut barrier-protective function) and *Desulfovibrionaceae_Desulfovibri* (LDA = 4.58) were significantly enriched. Control group (C) enriched in *Bacteroides_coprocola* (LDA = 4.61), Prevotellaceae (LDA = 4.48), *Ruminococcaceae_Subdoligranulum* (LDA = 4.70), and *Acidaminococcaceae_Phascolarctobacterium* (LDA = 4.87). These results demonstrate that resveratrol treatment (R1~R4) selectively modulated the abundance of functionally distinct microbial taxa, profoundly altering gut microbial composition and functional characteristics.

#### 3.5.6 Correlation analysis between intestinal microbiota and immune indicators

This study systematically explored the association between the gut microbial community and immune indicators in Cherry Valley ducks using genus-level redundancy analysis (CCA) and Spearman correlation heatmaps.

**Figure 10:**
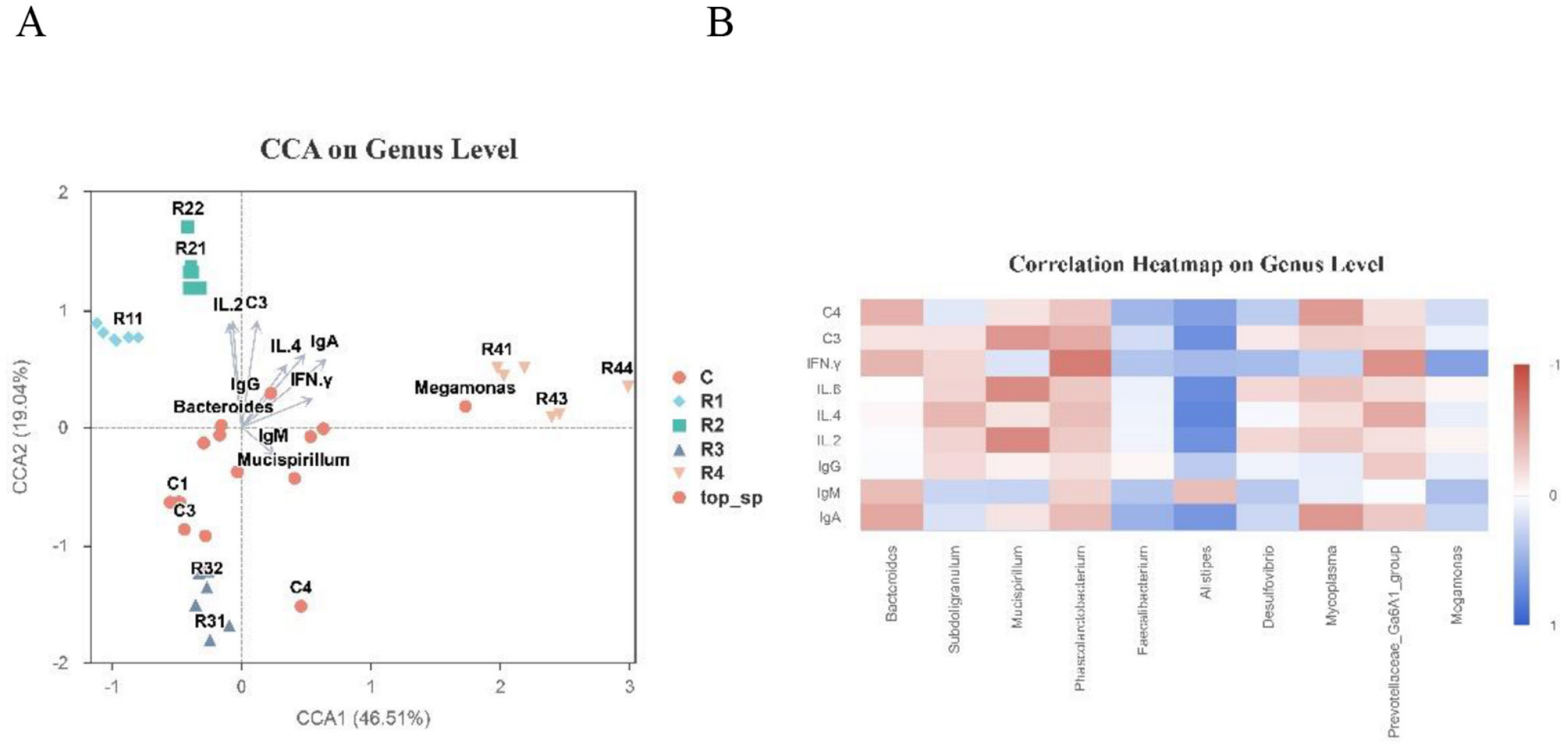
Correlation between intestinal microbiota and immune indicators with CAA on Genus Level **(A)** and Correlation Heatmap on Genus level **(B)**. Color bar maps heatmap data directly. Colors: bule means positive correlation vs. red means negative correlation. Arrows: environmental factors; length = correlation strength (longer = stronger). Angles: acute = positive correlation, obtuse = negative.

CCA Analysis Results (Figure 10A) show that the first two CCA axes (CCA1 and CCA2) explained 65.55% of the total community variation. Microbial distributions across treatment groups exhibited significant group-specific patterns. The characteristic microorganisms of R1 and R2 groups (e.g., R11, R21) showed significant positive correlations with pro-inflammatory cytokines (IL-2, IL-4) and immunoglobulins (IgA, IgG). Core microorganisms of the control group (e.g., Bacteroides, Mucispirillum) were concentrated around the coordinate center and closely associated with immunoglobulin indicators (IgM, IgG). Characteristic microorganisms of the R4 group (e.g., Megamonas) were located far from the center, showing weak correlations with immune indicators.

Spearman Correlation Heatmap (Figure 10B) shows that this further quantified the strength of associations between genera and immune indicators. *Alistipes* was significantly positively correlated with IL-2, IL-4, IL-6, IFN-γ, and complement factor C3 (*P* < 0.05). *Mucispirillum* showed positive correlations with IL-2 and IL-6. Bacteroides was significantly negatively correlated with IgA, IgM, C4, and IFN-γ (*P* < 0.05).

These results indicate that there are complex bidirectional associations between gut microbial community composition and immune indicators (cytokines, antibodies). Different resveratrol treatment groups, due to their distinct microbial community structures, formed unique “microbe-immune” association patterns, providing critical data to further elucidate their interaction mechanisms.

#### 3.5.7 PICRUSt community functional difference analysis

**Figure 11:**
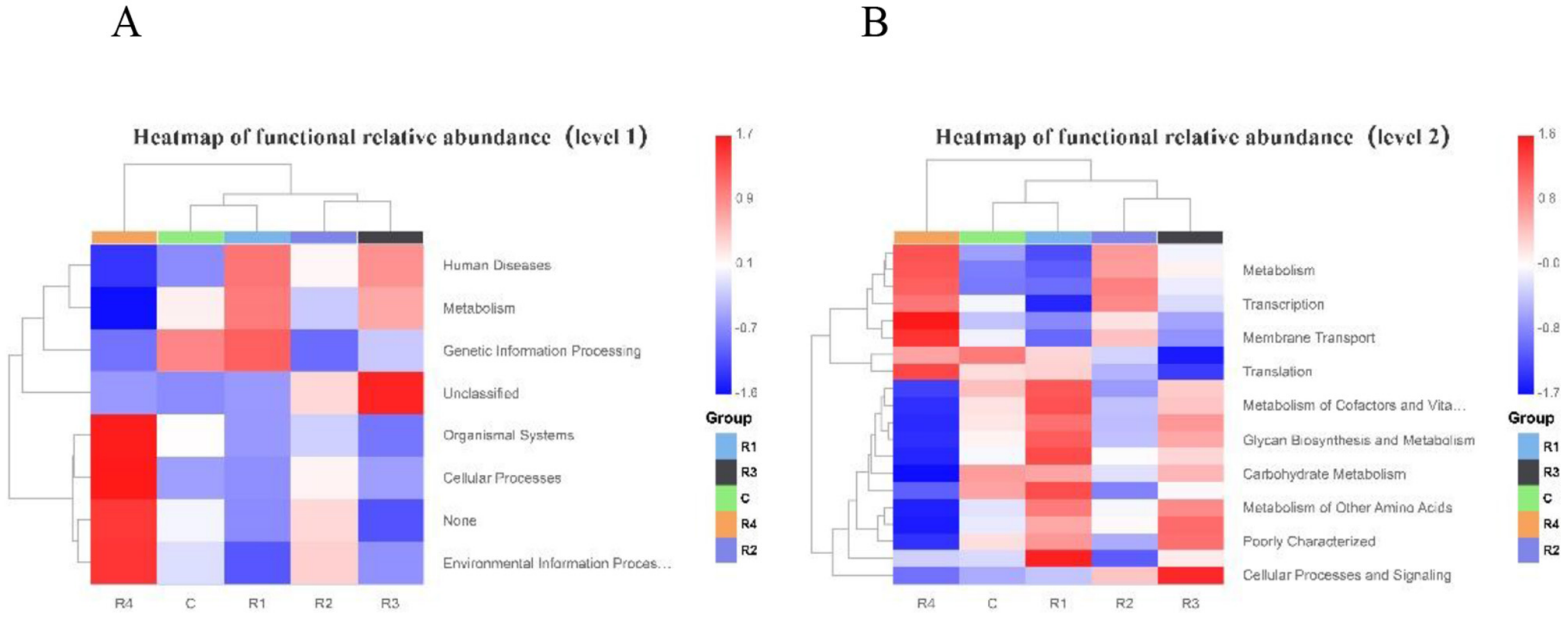
PICRUSt Heatmap of functional relative abundance on level 1 **(A)** and level 2 **(B)**.

This study systematically analyzed between-group differences in gut microbial functions between the control group and R1~R4 groups using primary and secondary classification functional relative abundance heatmaps. In the primary classification heatmap (Figure 11): The relative abundance of microbial taxa associated with the “Human Diseases” function was significantly higher in groups R1 and R3 compared to group R4. The abundance pattern of “Metabolism”-related taxa exhibited significant between-group divergence: R1 and R3 groups showed higher metabolic activity (higher abundance), while the R4 group had weaker metabolic function (lower abundance).

Notably, the R4 group displayed significantly enriched abundances of taxa related to “Organismal Systems,” “Cellular Processes,” and “Environmental Information Processing.”

Additionally, the R3 group had extremely high abundances of “Unclassified” taxa, suggesting it may carry more unknown functional genes. The secondary classification heatmap further refined functional differences: “Metabolism”-related taxa were enriched in groups R4 and R2 (higher abundance) but depleted in groups C and R1 (lower abundance). The abundance pattern of “Transcription”-related taxa showed significant divergence: low abundance in R1 and R3 groups, and moderate-to-high abundance in R2 and R4 groups. “Membrane Transport”-related taxa were more abundant in R2 and R4 groups but lower in R1 and R3 groups. For metabolic sub-functions such as “Metabolism of Cofactors and Vitamins,” “Biosynthesis and Metabolism of Sugars,” “Carbohydrate Metabolism,” and “Metabolism of Other Amino Acids,” the relative abundances of related taxa were significantly higher in R1 and R3 groups than in R4. In summary, both primary and secondary classification heatmaps clearly revealed divergence characteristics of abundance across functional categories (from macro to fine modules): R1 and R3 groups exhibited highly similar abundance patterns in certain functional modules (e.g., metabolic sub-functions), reflecting their functional association. Meanwhile, significant between-group differences in core functions (e.g., metabolism, transcription) provided critical evidence for in-depth exploration of the links between microbial functions and host phenotypes.

## 4 Discussion

### 4.1 Effects of resveratrol on growth performance

As a functional feed additive, resveratrol has demonstrated potential in alleviating the negative effects of heat stress in livestock and poultry farming. Previous studies have shown that it can effectively mitigate heat stress-induced reductions in feed intake and apparent digestibility of nutrients by regulating nutrient metabolism, improving gut function, and enhancing antioxidant stress responses, thereby promoting animal growth performance (Xie and Chen, 2023). Resveratrol has been reported to promote gut development and antioxidant function in broilers under heat stress, thereby improving their growth performance (Yujing et al., 2020). Further research by He et al. confirmed that supplementing resveratrol under heat stress conditions can positively regulate serum metabolic parameters and reduce tissue oxidative damage to improve the growth performance of broiler chicks (He et al., 2019a). However, the findings of this study are partially consistent with earlier reports, though some nuances are worth noting. While resveratrol did not significantly affect the average daily gain (ADG) or average daily feed intake (ADFI) of Cherry Valley ducks in this study, the feed-to-gain ratio (F/G) of the R1 group (supplemented at 14 days of age) was significantly lower than that of the control group (*P* < 0.05). This outcome diverges from the conclusion by Zhang et al. (2020) which suggested that “400 mg/kg resveratrol is ineffective” in broilers possibly reflecting differences in experimental conditions or animal models. At the same time, the observed improvement in F/G aligns with the trend reported by Li et al. (2023) where 250 mg/kg resveratrol enhanced the feed efficiency in Chinese local chickens.

These discrepancies may stem from species specificity: as a fast-growing aquatic bird, Cherry Valley ducks exhibit fundamental differences in nutrient metabolic pathways (e.g., energy allocation, intestinal absorption efficiency) compared to terrestrial poultry (e.g., broilers), leading to species-specific effects of resveratrol at the same dosage. Notably, although no significant changes in growth performance were observed in this study, the significant improvement in slaughter performance of R1~R4 groups suggests that resveratrol may exert its effects by optimizing nutrient allocation efficiency rather than directly promoting growth—a hypothesis consistent with the mechanism proposed by Meng et al. (2023). where resveratrol improves livestock and poultry production performance by regulating lipid metabolism, This study validates the potential of resveratrol in regulating the F/G ratio and slaughter performance in Cherry Valley ducks, with its effects likely dependent on species metabolic characteristics and supplementation timing, providing experimental evidence for the precise application of resveratrol in waterfowl farming.

### 4.2 Effects of resveratrol on immune function

The impact of resveratrol on the immune function of Cherry Valley ducks can be comprehensively analyzed from three aspects: immune organ development, serum immune indicators, and cytokine regulation. First, regarding immune organ indices, the thymus, spleen, and bursa of *Fabricius* are critical immune organs in poultry, and their relative weights (immune organ indices) directly reflect the body's immune status (Huang et al., 2004). In this study, the thymus and spleen indices of resveratrol-treated groups (R1~R4) showed an upward trend, though not reaching statistical significance (*P* > 0.05). However, considering the physiological characteristic of Cherry Valley ducks entering the immune decline phase at 42 days of age, this positive regulation still holds important physiological significance. Although not statistically significant, the upward trends in thymus and spleen indices suggest a potential delay in immune organ involution, which merits further investigation. This result aligns with the findings reported that resveratrol increased thymus and spleen indices in heat-stressed yellow-feathered broilers, validating its potential application in waterfowl. However, the influence of animal growth cycles and the natural changes in immune organs on the results should be noted (He et al., 2019b).

Second, for serum immune indicators, the complement system is a core component of the body's non-specific immunity, with C3 and C4 being key targets for immune regulation due to their high proportion and critical functions (Lee et al., 2025). This experiment found that the complement C3 content in the R2 group (supplemented at 21 days of age) was extremely significantly elevated (*P* < 0.0001), consistent with the conclusion by S He et al. that resveratrol increases C3 levels in heat-stressed broilers. The mechanism is hypothesized to involve activation of the macrophage TLR4 pathway, enhancing immune responses (Shi et al., 2025). However, no significant effects were observed on immunoglobulins (IgA, IgG, IgM)—markers of specific immunity—possibly due to differences in experimental dosage, animal species, or growth stages (He et al., 2019b).

Third, regarding cytokine regulation, IL-2 and IL-4 are key factors in balancing Th1/Th2 immunity. Their levels were significantly elevated in the R1 and R2 groups (supplemented at 14~21 days of age) (*P* < 0.01), indicating that resveratrol enhances the body's disease resistance by promoting Th1-type immune responses (cellular immunity). This result is consistent with the mechanism proposed by Liu S et al., where resveratrol exerts anti-inflammatory effects by inhibiting the TLR4/NF-κB pathway, further confirming that resveratrol supplementation for over 3 weeks has immunoregulatory and anti-inflammatory activity (Liu et al., 2024a).

In summary, resveratrol influences the immune function of Cherry Valley ducks through multiple pathways, including delaying immune organ degeneration, activating the complement system, and regulating Th1/Th2 balance. Its effects are closely related to the supplementation stage (e.g., the sensitive 21-day period), providing experimental evidence for the precise application of resveratrol in waterfowl farming.

### 4.3 Effects of resveratrol on intestinal microbiota

The regulation of intestinal microbiota by resveratrol and its functional impacts are the core focuses of this study. As the host's “second genome,” intestinal microbiota plays a critical role in maintaining poultry health and productivity by participating in nutrient digestion, immune development, and barrier maintenance (Dai et al., 2018; Vasaï et al., 2014; Wu et al., 2021). This study found that resveratrol significantly improved the gut health and immune status of Cherry Valley ducks by reshaping the intestinal microbiota structure, regulating metabolic functions, and influencing the microbiota-immune interaction.

#### 4.3.1 Intestinal microbiota diversity and structural remodeling

The Shannon index (diversity) and Chao1 index (richness) of the R2 and R4 groups were extremely significantly elevated (*P* < 0.001), indicating that resveratrol effectively increased microbial abundance and evenness. LEfSe analysis further revealed that the R4 group was specifically enriched with butyrate-producing *Faecalibacterium* (LDA = 4.15) and gut barrier-protective *Alistipes* (LDA = 4.32), while the *Bacteroidota* phylum was significantly increased in the R1 and R2 groups (+22%−35%), and *Deferribacterota* and opportunistic pathogens (e.g., *Prevotellaceae, Desulfovibrio*) were notably reduced. This pattern aligns with the findings in mice, where resveratrol increased *Bacteroidota* abundance (Chen et al., 2024); however, this study further observed that the elevation of *Bacteroidota* in R1 AND R2 groups was accompanied by a reduction in *Deferribacterota*. The *Bacteroidota* (*Bacteroidota*) in R1 and R2 groups was significantly increased, and its metabolites could activate intestinal epithelial CD4+ T cells, stimulate interleukin release, or reduce lipopolysaccharide (LPS) production, thereby alleviating inflammation (Yueqin et al., 2021). This explains why the R2 group exhibited extremely significant upregulation of IL-2 and IL-4—likely driven by microbiota-induced CD4+ T cell activation (He et al., 2023). Conversely, the observed reductions in *Prevotellaceae* (known short-chain fatty acid producers) and *Desulfovibrio* (sulfate-reducing bacteria) in both the R1 and R2 groups suggest a potential mechanism by which resveratrol may influence gut health. While not directly measured in this study, previous research indicates that metabolites from these bacterial groups may inhibit the NF-κB pathway and alleviate H_2_S-induced damage to intestinal epithelial mitochondrial function, respectively (Scardino et al., 2025; Singh et al., 2023). It is worth noting that the association with SCFA mechanisms remains speculative in the present context, but the suppression of these bacterial taxa could theoretically contribute to reduced inflammation and enhanced intestinal barrier function.

#### 4.3.2 Microbiota-immune interaction mechanisms

CCA analysis demonstrated that microbiota composition dominated immune responses. Microbial clusters in R1 AND R2 groups (e.g., R11, R21) were positively correlated with IL-2, IL-4, IgA, and IgG, suggesting that early supplementation (14~21 days of age) activated the Th2 immune pathway by enriching specific microbiota. The Prevotellaceae enriched in the R2 group may regulate the NF-κB/MAPK signaling pathway via butyrate metabolism, inhibiting Pseudomonas aeruginosa gene expression and promoting IL-6 secretion (Bertelsen et al., 2021). Additionally, the upregulated short-chain fatty acid (SCFA) synthesis genes in Faecalibacterium led to increased butyrate levels, which modulated gut lumen pH, inhibited NF-κB activation, and upregulated IL-10 expression, synergizing with elevated IL-2/IL-4 to exert anti-inflammatory effects (Munukka et al., 2017). Correlation analysis further confirmed that *Alistipes* was significantly positively correlated with IL-2 and IL-4 (*r* > 0.6), potentially enhancing mucosal immunity via tryptophan metabolism to produce indole derivatives (Liu et al., 2024b); in contrast, the negative correlation between *Bacteroides* (*Bacteroides*) and IgA/IgM (*r* < −0.5) challenged the traditional view that all *Bacteroidetes* are beneficial, suggesting their functions may be regulated by metabolite type (Zhu and Ding, 2024).

#### 4.3.3 Functional regulation of intestinal microbiota

As the host's “second genome,” the functional diversity of gut microbiota influences poultry health through metabolic and immune regulation (Hussain and Bhattacharyya, 2024). PICRUSt functional heatmaps revealed differentiated regulation of gut microbiota function between resveratrol-treated groups (R1~R4) and the control group: At the primary classification level, “Human Diseases” function abundance was significantly higher in R1&R3 groups than in R4, while “Metabolism” function activity (R1&R3) contrasted with “Organismal Systems” and “Cellular Processes” enrichment in R4. At the secondary classification level, metabolism-related taxa were enriched in R2&R4 groups; genetic transcription and membrane transport were active in R2&R4 groups; and carbohydrate metabolism was more pronounced in R1&R3 groups. Consistent with previous mammalian studies (e.g., mice, broilers), resveratrol promoted lipid and carbohydrate metabolism (Chen et al., 2024; Jie et al., 2024; Qin et al., 2025). By reshaping microbiota function (metabolism, homeostasis), resveratrol influenced Cherry Valley duck health, providing theoretical support for precision nutrition in poultry.

This study is the first to systematically reveal the mechanism by which resveratrol improves gut health in Cherry Valley ducks via the “microbiota structural remodeling-metabolic function regulation-immune response activation” axis, offering metabolic-level theoretical evidence for precision nutrition in waterfowl. However, the study did not detect microbiota metabolites (e.g., SCFAs, tryptophan derivatives) or correlate microbiota changes with disease resistance (e.g., challenge tests). Future studies should employ metabolomic and transcriptomic analyses to elucidate the underlying molecular mechanisms and substantiate the functional claims regarding the synergistic effects of the 21-day resveratrol supplementation.

## 5 Conclusion

Using Cherry Valley ducks as a model, this study preliminarily explored the effects of resveratrol supplementation at different stages. The results suggest that resveratrol may improve slaughter performance, enhance nutrient utilization efficiency, and elevate humoral immunity markers (IgA, complement C3) and cytokine expression (IL-2, IL-4). It also appeared to modulate gut microbiota composition by increasing beneficial bacteria and suppressing potential pathogens. Notably, supplementation initiated at 21 days of age (R2 group) showed relatively stronger effects on slaughter traits and immune-microbiota coordination. However, these observations are constrained by fixed dosage, selected endpoints, and lack of metabolite validation. The findings tentatively support a potential “microbiota-immune-metabolism” regulatory axis, offering preliminary insights for sustainable duck production.

## Data Availability

The data presented in the study are deposited in the NCBI repository, accession number PRJNA1300597.
